# Rapid and sensitive detection of botulinum toxin type A in complex sample matrices by AlphaLISA

**DOI:** 10.3389/fpubh.2022.987517

**Published:** 2022-10-20

**Authors:** Liwen Zhang, Qingyu Lv, Yuling Zheng, Shan Gao, Wenhua Huang, Peng Liu, Decong Kong, Ye Wang, Yunzhou Yu, Yongqiang Jiang, Hua Jiang

**Affiliations:** ^1^Anhui Medical University, Hefei, China; ^2^State Key Laboratory of Pathogen and Biosecurity, Institute of Microbiology and Epidemiology, Academy of Military Medical Sciences, Beijing, China; ^3^Beijing Institute of Biotechnology, Beijing, China

**Keywords:** botulinum toxin type A, AlphaLISA, complex sample matrices, food safety, food poisoning

## Abstract

Botulinum toxin A(BoNT/A) is a neurotoxin produced by the bacteria *Clostridium botulinum*, which can cause serious food poisoning and is recognized as a potential biological warfare agent. BoNT/A is does not degrade easily and can remain in the complex matrix for a long time. Meanwhile, the poisonous dose of botulinum toxin exceptionally low and intravenous human lethal doses estimated at 1-3 ng/kg. Therefore, sensitive and accurate detection methods suitable for testing a wide range of complex samples are urgently needed. To this end, the “amplified luminescent proximity homogeneous assay linked immunosorbent assay” (AlphaLISA) was established for the detection of BoNT/A and its detection efficacy in plasma, beverage, food, and other complex samples was evaluated. The results showed that this method can very effectively resist matrix interference. The detection time is rapid, reaching a detection limit for all samples of up to 0.1 ng/mL in only 30 min. BoNT/A can also be accurately detected in vomit samples of patients with clinical food poisoning. This study demonstrates that AlphaLISA is an effective tool for the detection of BoNT/A in complex samples and can potentially be developed for commercial use in the future.

## Introduction

Botulinum toxin (BoNT) is a neurotoxin produced by the bacteria *Clostridium botulinum* and exists as a non-toxic precursor in bacteria. Protease treatment turns this precursor into an active toxin, composed of a heavy (H) chain (~100 kD) and a light (L) chain (~50 kD). The H chain can bind to neuron receptors and the L chain has protease activity and cleaves proteins necessary for nerve signal transmission. As such, BoNT has caused significant neurotoxicity and has been included on the highest risk (category A) biothreat agents by the US Centers for Disease Control and Prevention (CDC). BoNT can be divided into 7 serotypes (A-G), among which BoNT/A, B, E, and F are the primary ones responsible for human poisoning. Type A is the most toxic (1), and could potentially be used in terrorist attacks and biological warfare. Besides neurotoxicity, BoNTs also kills animals by entering the digestive system and ultimately weakening the vital organs. The exceptionally low lethal dose of BoNTs is estimated from primate studies to be 1 μg/kg body weight when received orally, 10 ng/kg by inhalation, and 1 ng/kg when received intravenously or intramuscularly (2–5).

BoNTs exist in complex food and environmental sample matrices, the content is very low and can remain stable for a long time. Botulism can be acquired by humans in several different ways. They are foodborne botulism, infant botulism, wound botulism, and inhalation botulism or other types of intoxication (https://www.who.int/news-room/fact-sheets/detail/botulism). All kinds of botulism can be fatal, so rapid and serotype specific detection of botulism is especially important for epidemic evidence, therapeutic applications, and food safety (6). Likewise, as a potential biological warfare agent, detection of toxins in variety of samples is necessary for countering the threat of potential bioterrorist attacks.

Presently, the United States FDA relies on mouse lethality tests as the standard assay for detecting the potency of BoNTs in clinical and food samples. This method is sensitive, but expensive and time consuming (1–7 days), lacks standardization, and can't meet rapid demands (3). Other detection methods include HPLC, MS, and ELISA (7–9). However, while these methods are suitable for detection of toxins in buffer or purified toxins, most of them have not been proven suitable for detection in complex matrix samples such as food, serum, and more.

Detection methods for BoNTs in complex samples have been reported, such as MALDI-TOF and LC-MS/MS, but these methods require the toxin be separated from complex samples by high affinity, serotype specific, monoclonal antibodies and identified by mass spectrometry (10–12). The AlphaLISA method is homogeneous and requires less time to perform. Due to the short lifespan of singlet oxygen in solution, the background signal of this method is relatively low. From our previous research on this method (13, 14), we found that matrix interference was relatively low. We evaluated the efficacy of this detection method on botulism contaminated samples by testing a wider range of sample types. Clinical patient samples were also included to further evaluate the detection efficacy of the AlphaLISA method. The results showed that the AlphaLISA method could quickly and accurately identify BoNT/A in many different sample types.

## Materials and methods

### Antigens, antibodies, and reagents

BoNT/A was made in our laboratory. The BoNT/A antibody pairs used in this study include a monoclonal antibody and a polyclonal horse antibody, both of which were generously provided by Professor Yunzhou Yu (1). The Eu acceptor beads and streptavidin donor beads were from PerkinElmer (Waltham, MA, USA). The buffer used in the reaction was 25 mM HEPES (pH 7.4, 0.1% casein, 1 mg/mL dextran-500, 0.5% Triton X-100, and 0.05% proclin-300). The horse antibody was conjugated with acceptor beads as previously reported (14). Conjugation of the monoclonal antibody to biotin was done using the EZ-Link^®^ Sulfo-NHS-LC-Biotinylation kit (Thermo Scientific, Rockford, IL, USA).

### AlphaLISA experiment

20 μL of acceptor beads (62.5 μg/mL) and biotin-labeled antibody (0.5 μg/mL) mixture, and 5-μL of specimen was added to into the wells of a 96 well plate. The 25-μL Mixture Was Incubated at 37 °C for 15 min, and then 25 μL of donor beads (25 μg/mL) were added. After incubation for an additional 10 min at 37 °c, the signal was read by a microplate reader (SpectraMax™ I3, Molecular Devices, San Jose, CA, USA). The cutoff value was the mean of 0 ng/mL + 3 standard deviations (SD).

### ELISA experiment

2 μg/mL of anti BoNT/A horse antibody was used to coat the wells a 96-well microtiter plate overnight at 4 °C. The wells were then blocked with 200 μL of PBST (PBS With 0.5% Tween-20) containing 3% BSA for 2 h. BoNT/A at different concentrations was added and incubated at 37 °C for 30 min. The supernatant was discarded, and the plate washed thrice with PBST. The biotin-labeled detection antibody was added and incubated for 30 min at 37 °C. After washing with PBST, horseradish peroxidase-streptavidin (1:8000 dilution, Thermo Scientific™) was added and the mixture incubated for 30 min at 37 °C. After the substrate was added, the signal was recorded by a SpectraMax I3(Molecular Devices, Sunnyvale, CA, USA) at 450 nm. The cutoff value was the OD_450_ 0ng/mL + 3SD.

### Liquid spiked specimen detection

Healthy adult plasma samples were collected from the Fifth Medical Center, Chinese PLA General Hospital (Former 307th Hospital of the PLA). The plasma was diluted 2-fold by buffer or left undiluted. BoNT/A was added to make plasma spiked specimens. BoNT/A was added to whole milk with or without double dilution by buffer to make milk spiked specimens. For juice, pretreatment included centrifugation at 8000 rpm/min for 10 min, twice, to remove particulate material. The supernatant was taken and then BoNT/A was added to the juice supernatant or water to make spiked specimens. For all tests, the cutoff value was the mean of 0 ng/mL + 3 standard deviation (SD).

### Solid spiked specimen detection

For solid specimen pretreatment, 0.4 g of specimen was weighed aseptically and added to a 1.5 mL EP tube. 1 mL of buffer was added and the specimen homogenized with a homogenizer for 10 min at 60 HZ. After homogenization, the specimen was centrifugated at 8000 rpm/min for 10 min. The supernatant was taken as the matrix. Buffer was added again to make 5, 10, and 20% (w/v) dilutions. BoNT/A was added to make spiked specimens at different concentrations. For all tests, the cutoff value was the mean of 0 ng/mL + 3 standard deviations (SD).

### Clinical samples detection

Three vomitus and two stool specimens were obtained from botulism patients in the Fifth Medical Center, Chinese PLA General Hospital (Former 307th Hospital of the PLA). All samples were from severe food poisoning patients. The specimens were centrifugated at 8000 rpm/min for 10 min, the supernatant was taken, and the pH was adjusted to 7.0 by 800 mM NaOH. At the same time, clinical vomit samples were tested in parallel with commercial colloidal gold reagent. For the two stool samples, DNA was extracted using Macherey-Nagel™ NucleoSpin™ DNA Stool kit (MN, Düren, Germany). DNA were used as template for qPCR detection. Specific primers for BoNT/A, /B, /E and /F are listed in Supplementary Table S1. The qPCR mix used was TaqMan™ Universal Master Mix II, with UNG (Thermo Fisher). All clinical samples were retrospective.

### Statistical analysis

Data are presented as mean ± standard deviation (SD). The student's *t*-test was used to compare two groups. *P* < 0.05 was considered statistically significant.

## Results

### Optimization

**Figure 1:**
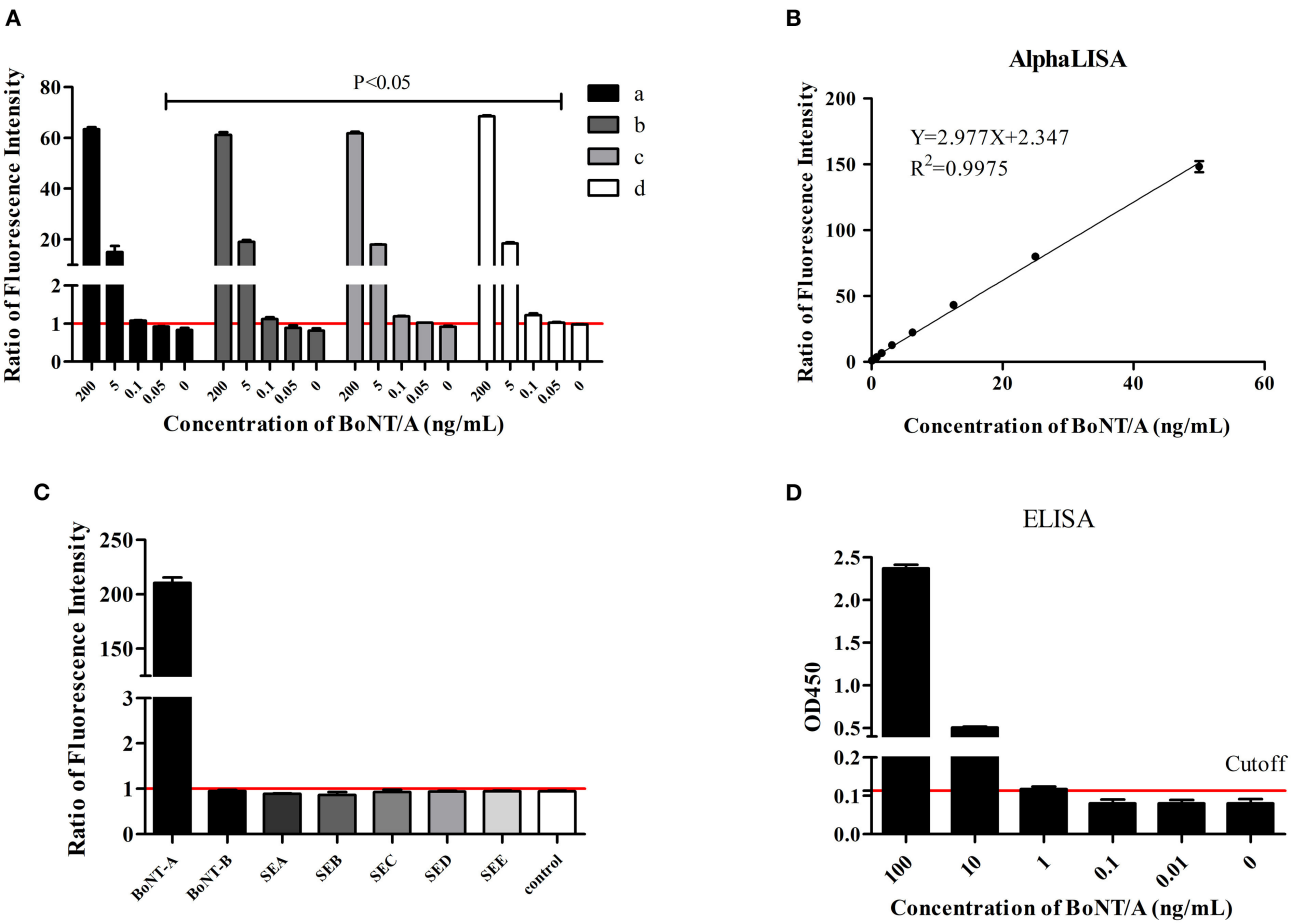
BoNT/A detection in buffer. Ratios of fluorescence intensity were the measured values for samples at different concentrations divided by the cutoff values. The cutoff value was the mean of 0 ng/mL + 3 SD. **(A)** Evaluation of the optimal concentration of horse antibody conjugated acceptor beads, biotinylated antibody, and streptavidin donor beads. (a) Acceptor beads at 100 μg/mL, biotinylated antibody at 1 μg/mL, donor beads at 40 μg/mL; (b) Acceptor beads at 100 μg/mL, biotinylated antibody at 0.5 μg/mL, donor beads at 40 μg/mL; (c) Acceptor beads at 62.5 μg/mL, biotinylated antibody at 1 μg/mL, donor beads at 33.3 μg/mL; (d) Acceptor beads at 62.5 μg/mL, biotinylated antibody at 0.5 μg/mL, donor beads at 25 μg/mL. **(B)** The linear range of AlphaLISA for BoNT/A detection. **(C)** Specificity evaluation of AlphaLISA. The reaction buffer was as control. **(D)** ELISA test. Cutoff value was the OD_450_ 0 ng/mL + 3SD.

For AlphaLISA detection system, the right proportions of acceptor beads, biotin-labeled antibody, and donor beads are needed to maximize its sensitivity and specificity (13, 14). Based on our previous experience, concentrations of 62.5 μg/mL and 100 μg/mL of horse polyclonal antibody coupling acceptor beads, 0.5 μg/mL and 1 μg/mL of biotinylated monoclonal antibody, and 25 ug/ml, 33.3 μg/mL, and 40 μg/mL of streptavidin donor beads were evaluated. After repeated evaluation of the detection limit of BoNT/A in buffer, the results showed that the detection sensitivity was highest with 62.5 μg/mL horse polyclonal antibody coupling acceptor beads, 0.5 μg/mL biotinylated monoclonal antibody, and 25 μg/mL streptavidin donor beads (Figure 1A). All following experiments used this reaction condition.

### Sensitivity and specificity

Based on the above optimized detection conditions, BoNT/A diluted in buffer in a 2-fold gradient from 0.025 ng/mL to 100 ng/mL was used to test the sensitivity. Four parameters were used to fit the concentration and signal values. The results showed that the limit of detection (LOD) is 0.05 ng/mL for BoNT/A in buffer and the linear range was 0.05 ng/mL to 50 ng/mL (Figure 1B). ELISA tests with the same antibody demonstrated the sensitivity to be 1 ng/mL (Figure 1D).

In evaluating the specificity, 100 ng/mL BoNT/A, BoNT/B, and staphylococcal enterotoxin (SEA, SEB, SEC, SED and SEE) were included. The results showed that the AlphaLISA for BoNT/A did not cross-react with these other six toxins (Figure 1C).

### Precision (CV value)

BoNT/A was diluted to 0.08, 6, and 20 ng/mL by buffer. The detection of BoNT/A at different concentrations was repeated 12 times. The results showed that the CV values of the three concentrations were all < 6%, which indicates that the repeatability of this method is very good (Table 1).

**Table 1:** Reproducibility of botulinum toxin tests (*n* = 12).

**Concentration**	**Mean of fluorescence intensity**	**Standard deviation**	**Coefficient of variation**
20 ng/mL	352186	6640	1.89%
6 ng/mL	116869	3353	2.87%
0.08 ng/mL	6107	344	5.63%

### Spiked and clinical specimen detection

**Figure 2:**
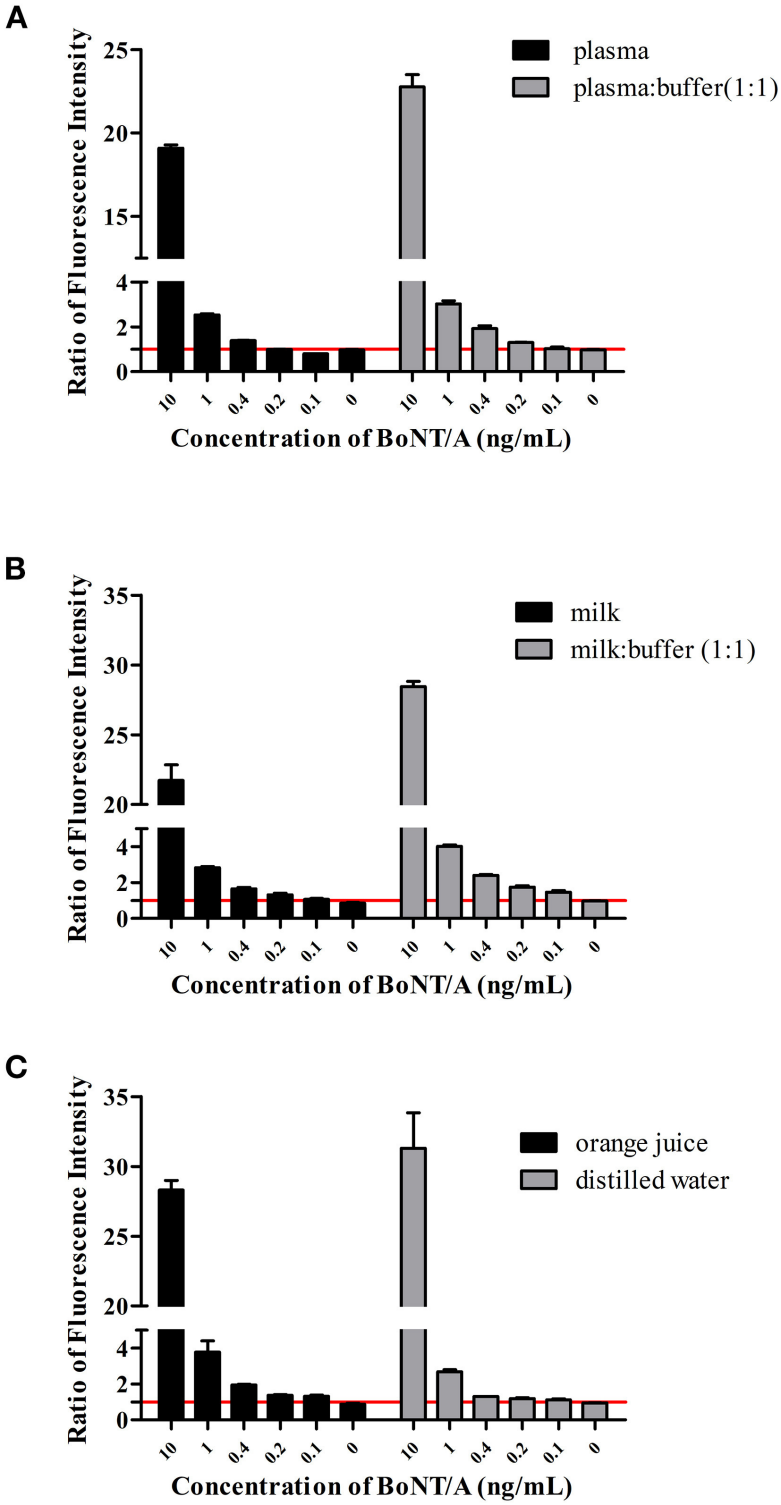
AlphaLISA detection in the liquid simulation samples. Detection of BoNT/A in plasma **(A)**, milk **(B)**, orange juice, and water **(C)** simulated samples. Ratios of fluorescence intensity were the measured values for samples at different concentrations divided by the cutoff values. The cutoff value was the mean of 0 ng/mL + 3 SD.

Previous studies have shown that AlphaLISA tolerates the sample matrix well (13, 14). Here we test additional sample types, diluted or undiluted, to evaluate the influence of different sample matrices on the detection ability of this method. For detection in plasma simulation samples, we prepared undiluted and 2-fold diluted samples. The results showed that if the plasma was not diluted, the LOD only reached 0.4 ng/mL, while for diluted plasma the LOD reached 0.1 ng/mL (Figure 2A). The difference may be due to interference by proteins or lipids in the plasma (15).

For undiluted milk samples, the signal value of each concentration was lower than that of the 2-fold diluted simulated milk sample. Dilution had no effect on detection sensitivity and the LOD under both conditions reached 0.1 ng/mL, however the signal and S/N (signal/noise) declined for the undiluted milk samples (Figure 2B).

The pH values of the orange juice and distilled water samples were 3.0 and 7.0, respectively. To evaluate the effect of acidity on detection, BoNT/A was directly added to the supernatant of centrifugally clarified orange juice and distilled water samples. The results showed that the LOD of BoNT/A reached 0.1 ng/mL in both samples (Figure 2C) indicating acidity has little influence on detection.

**Figure 3:**
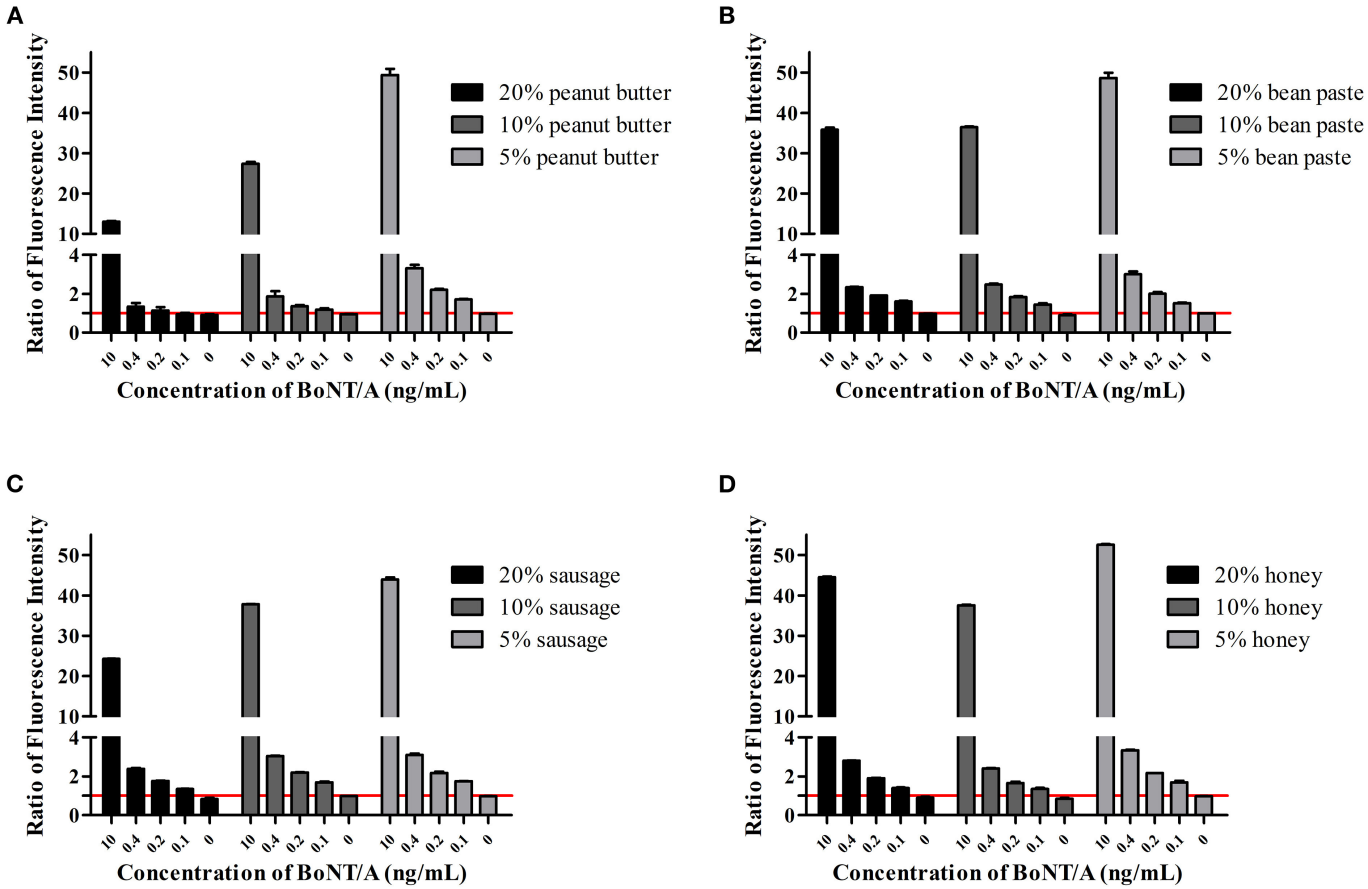
AlphaLISA detection in the solid simulation samples. Detection of BoNT/A in peanut butter **(A)**, bean paste **(B)**, sausage **(C)**, and honey **(D)** simulated samples. Ratios of fluorescence intensity were the measured values for samples at different concentrations divided by the cutoff values. The cutoff value was the mean of 0 ng/mL + 3 SD.

For solid simulated samples such as peanut butter, bean paste, sausage, and honey, the samples were first treated, diluted with buffer, and then BoNT/A was added. The results show that at a 20% dilution, the LOD for peanut butter is 0.2 ng/mL, and the others are 0.1 ng/mL. At 5 and 10% dilutions, the LOD of all solid simulation samples reached 0.1 ng/mL. The S/N of the 5% dilution was the highest (Figure 3). It can be inferred that dilution is necessary for solid samples.

**Figure 4:**
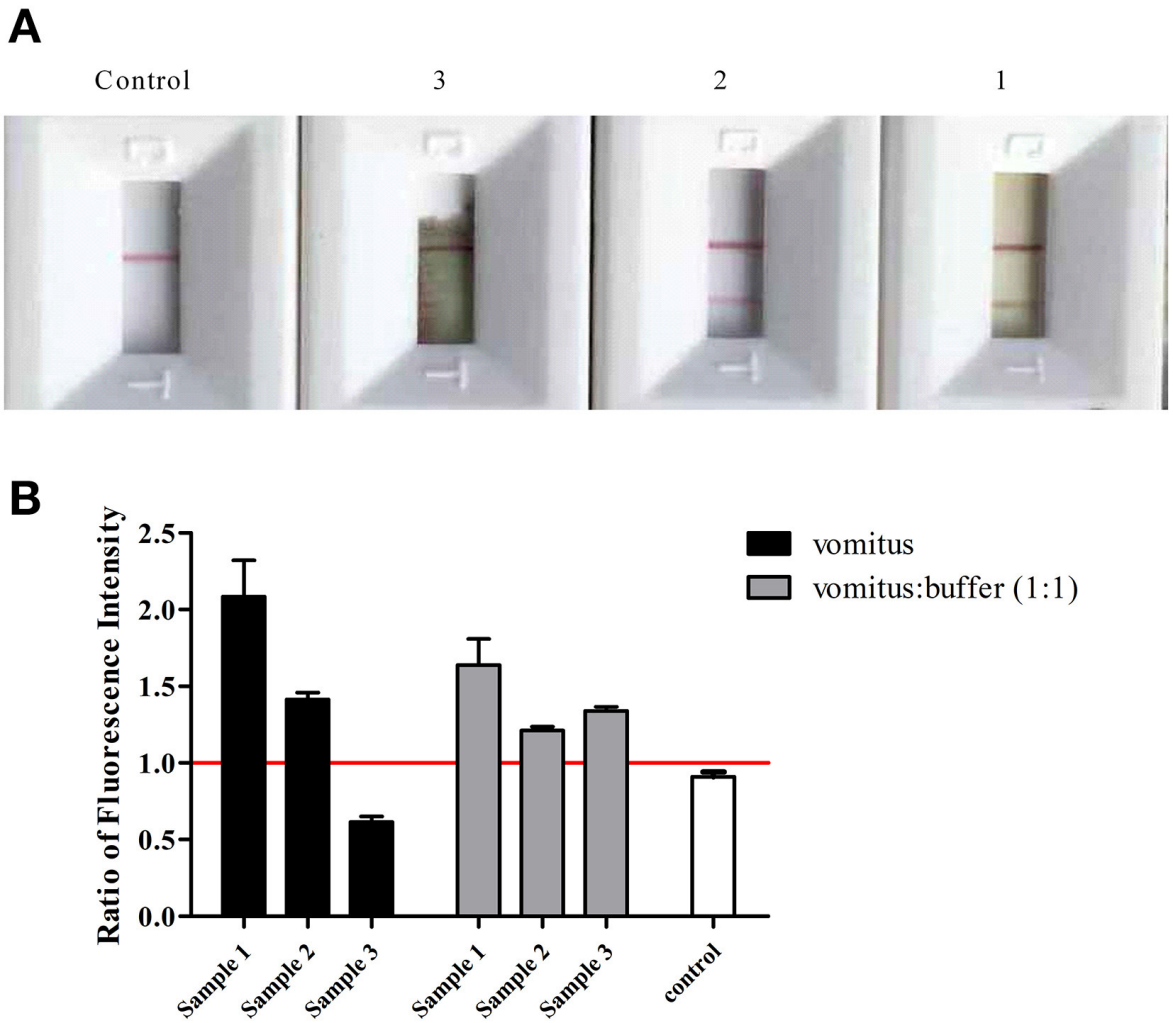
Detection in clinical specimens. **(A)** Colloidal gold detection in clinical specimens. **(B)** AlphaLISA detection results for clinical specimens. Ratios of fluorescence intensity were the measured values for samples at different concentrations divided by the cutoff values. The cutoff value was the mean of control + 3SD. The reaction buffer was as control.

For detection in clinical samples, such as patients' vomit, we first used commercial colloidal gold strips (Beijing KingHawk Pharmaceutical Co., LTD, Beijing, China.) to test the samples. The results showed that both No. 1 and No. 2 samples were positive, but No. 3 was suspected (Figure 4A). From the results of No. 3, the chromatography is incomplete, which may relate to the viscosity of the vomit sample; after centrifugation, we found that the viscosity of sample 3 was higher than of samples 1 and 2. Considering that the sensitivity of colloidal gold is 10 ng/mL, to avoid false negatives after dilution, we did not dilute and retest the No. 3 sample. For the undiluted samples tested by AlphaLISA, No. 1 and No. 2 were positive and sample No. 3 was negative. With 2-fold dilution, the positive signal value increased. All three diluted samples were BoNT/A positive, which was consistent with the clinical diagnosis (Figure 4B).

## Discussion

In this study, the AlphaLISA method was evaluated for rapid and accurate identification of BoNT/A in a variety of complex sample matrices. For BoNT/A in buffer, the detection sensitivity can reach 0.05 ng/mL. For liquid, solid, and clinical simulation samples, the detection sensitivity reached 0.1 ng/ml, which is much lower than the toxic dose. The results of clinical sample tests showed BoNT/A could be accurately detected in vomit samples from patients experiencing botulism poisoning. As a potential diagnostic antibody for BoNT/A, it has good specificity and does not exhibit cross reaction with enterotoxins and BoNT/B. It could clearly distinguish between BoNT/A and BoNT/B, which is very important for botulism detection, because different serotypes of botulism need to be neutralized with specific antisera (16, 17).

The ability to detect BoNTs in food is necessary to identifying contaminated food and prevent outbreaks of botulism. However, foods are often protein-rich, low pH, high fat, or very viscous. The highly variable natures of these different food matrices pose a challenge to BoNTs detection (3, 12). For antigen-antibody reactions, it has previously been reported that the acidity of freshly squeezed orange juice and the fat or casein in milk can affect antigen-antibody binding, which could negatively affect the assay (15). To reduce the effects of the food matrix, some studies isolated, tested, and quantified toxins from food samples (3, 16). In this study, the AlphaLISA method was not only found to be sensitive and rapid, but also showed a certain tolerance to a variety of different sample type matrices. For serum or plasma samples, dilution could improve the detection sensitivity from 0.4 ng/mL to 0.1 ng/mL. It may be that components in the plasma have a blocking effect on the signal. For food samples, although dilution did not improve the detection sensitivity, it increased the signal/noise value. We compared the composition tables of milk, peanut butter, bean paste, sausage, honey, and orange juice samples and found that, excluding orange juice, the protein and lipid contents were high for the others, which might also affect the detection signal. Therefore, it is recommended that such samples be diluted before testing.

In this study, we found that the acidity of orange juice (pH 3) had little influence on detection by AlphaLISA. Without dilution or adjusting the pH, the sensitivity still reached 0.1 ng/mL. This indicates that the AlphaLISA detection system could at least withstand an acidity of pH 3. However, compared with orange juice, the pH of gastric juices 0.9–1.5, much lower than that of orange juice. Because BoNT substrate neurotoxin-associated proteins (NAPs) can protect the toxin from digestion when exposed to pepsin at pH 2, the matrix proteins are digested and the toxin complex remains intact (16), which is also one reason why the toxin can be detected in vomit by mass spectrometry. Here, however, since we did not try a lower pH than orange juice, we did not determine the detection ability of AlphaLISA for lower pH samples. The clinical vomit samples collected in this study are sticky, complex, and acidic. To remain consistent with sample processing of commercial colloidal gold detection, pretreatment includes centrifugation and pH adjustment. However, even after centrifugation, the supernatant viscosity of sample 3 was still high, which may explain why the colloidal gold test result of undiluted sample No. 3 was “suspected”. Meanwhile, the high viscosity also greatly impacts the detection of AlphaLISA due to the inherent stickiness of the matrix which inhibits toxin binding to capture beads and/or biosensors. Christina CT et al. tried to detect botulinum toxin in undiluted liquid eggs but failed. Therefore, it is proposed that before adding BoNT/A, liquid eggs must be diluted 1:10 with detection buffer (5).

Additionally, we collected two BoNT/B positive stool samples. In these, we detected the BoNT/B gene by qPCR (Supplementary Figure S1), but did not detect the BoNT/A, /E, /F gene. We then used the established AlphaLISA method to diagnose the sample, and no BoNT/A was detected, which confirmed the detection method dose not cross react with BoNT/B.

In conclusion, through the detection of BoNT/A in different sample type matrices, we found that the AlphaLISA method was effective in and resisted interference by a wide range of matrix types, and it exhibited good sensitivity and specificity for BoNT/A. Based on our previous detection of enterotoxin B and T2 toxin, we believe that AlphaLISA has great potential to become a rapid toxin detection method for foods in the future.

## Data availability statement

The original contributions presented in the study are included in the article/Supplementary material, further inquiries can be directed to the corresponding authors.

## Author contributions

HJ, YJ, and QL contributed to study conception and design. LZ, QL, YZ, and WH performed the experiments. SG, PL, DK, YW, and YY contributed reagents and materials. HJ and QL wrote the manuscript. All authors participated in interpreting the results, read, and approved the final version of this manuscript.

## Funding

This work was supported by grants from the State Key Laboratory of Pathogen and Biosecurity (SKLPBS2119).

## Conflict of interest

The authors declare that the research was conducted in the absence of any commercial or financial relationships that could be construed as a potential conflict of interest.

## Publisher's note

All claims expressed in this article are solely those of the authors and do not necessarily represent those of their affiliated organizations, or those of the publisher, the editors and the reviewers. Any product that may be evaluated in this article, or claim that may be made by its manufacturer, is not guaranteed or endorsed by the publisher.
