# Supplementary material for: Rapid and sensitive detection of botulinum toxin type A in complex sample matrices by AlphaLISA
Source: Front Public Health. 2022 Oct 20;10:987517. doi: 10.3389/fpubh.2022.987517 (PMC9632490; doi:10.3389/fpubh.2022.987517)
Supplement: Supplementary file 1 [file Table_1.docx]

**Supplementary Materials：**

**TABLE S1** **|** Primers for botulinum toxin detection in stool samples.

| Toxins | Sequence (5’-3’) |
| --- | --- |
| BoNT/A | F: GGAGTCACTTGAAGTTGATACAAATC |
|  | R: GCTAATGTTACTGCTGGATCTGTAG |
|  | P: TCTTTTAGGTGCAGGCAAATTT |
| BoNT/B | F: GATGAACAGCCAACATATAGTTGTCA |
|  | R: GTTTCCTTTTTACCTCTTTTAAGTACCATT |
|  | P: TGATGAKATAGGATTGATTGGTATTCA |
| BoNT/E | F: CTATCCAAAATGATGCTTATATACCAAA |
|  | R: GGCACTTTCTGTGCATCTAAATA |
|  | P: ATGATTCTAATGGAACAAGTGATATAGAACAACATGATGT |
| BoNT/F | F: GCAATATAGGATTACTAGGTTTTCATTC |
|  | R: GAAATAAAACTCCAAAAGCATCCATT |
|  | P: TTGGTTGCTAGTAGTTGGTATTATAACAA |

**FIGURE S1** BoNT/B gene confirmed by qPCR.
